# The Inhibition of Group II Innate Lymphoid Cell Response by IL-27 in Allergic Rhinitis

**DOI:** 10.1155/2020/6661524

**Published:** 2020-12-16

**Authors:** Xi Luo, Qingxiang Zeng, Yan Li, Yiquan Tang, Wenlong Liu, Renzhong Luo

**Affiliations:** Department of Otolaryngology, Guangzhou Women and Children's Medical Center, Guangzhou Medical University, Guangzhou, China

## Abstract

**Objectives:**

Interleukin-27 (IL-27) has been reported to inhibit type 2 T helper cell (Th2) response in allergic rhinitis (AR). However, its effects on group II innate lymphoid cells (ILC2) in AR are not fully understood.

**Methods:**

Nineteen patients with AR and nineteen controls were enrolled in this study. The effects of IL-27 on ILC2 differentiation and function as well as the regulation of the IL-27 receptor (IL-27R) were analyzed by tritiated thymidine incorporation, enzyme-linked immunosorbent assay (ELISA), and real-time polymerase chain reaction (PCR), respectively. AR mice were used to confirm the role of IL-27 *in vivo*.

**Results:**

The serum IL-27 protein expression in AR patients was significantly lower compared with controls. IL-27 decreased the ILC2 proliferation and type II cytokine secretion through the interaction with IL-27R. IL-27 also inhibited systemic and nasal ILC2 response of AR mice.

**Conclusion:**

IL-27 inhibited the proliferation and function of ILC2 in AR, implying that IL-27 may be used as new treatment target in AR.

## 1. Introduction

Allergic rhinitis (AR), one of the most common diseases in otorhinolaryngology, affects 10%-20% of the whole population [1]. Clinical symptoms of AR included nasal obstruction, itchy nose, sneezing, and runny nose, which affect the quality of life of patients and consume huge social and medical costs.

IL-27, which belongs to the IL-12 family, is composed of the p28 and Epstein-Barr virus-induced gene 3 (EBI3) subunits. The IL-27 is mainly produced by activated antigen presenting cells (APCs) [2, 3]. The IL-27 receptor consists of glycoprotein 130 (gp130) and WSX-1 [4]. Previous studies showed that IL-27 can induce type 1 T helper cell (Th1) inflammation, inhibit Th17 response, and induce IL-10-producing Foxp3^−^CD4 T cells [5–7]. In Th2-related allergic diseases, IL-27 was reported to inhibit Th2 cell differentiation both *in vitro* and *in vivo* and type II cytokine production from Th2 effector cells [8–10]. Consistently, enhanced allergic inflammation is found in mice deficient in IL-27 receptor *α* (IL-27R*α*) [11].

Group II innate lymphoid cells (ILC2), distributed in various organs, play key roles in the early stage of immune response [12]. Studies have found that the frequency of ILC2 in the peripheral blood of AR patients sensitized to pollen increased in pollen season [13]. Moreover, Fan et al.'s study found that ILC2 may exert different roles in AR subtypes mediated by dust mites and mugwort [14]. Moro et al. and Mchedlidze et al.'s studies had proven that IL-27 treatment inhibited ILC2 proliferation and cytokine production and significantly reduced their accumulation in mouse models [15, 16].

In this study, we aimed to explore the role of IL-27 on ILC2 differentiation and type II cytokine production using cell and animal models.

## 2. Methods

### 2.1. Subjects

All 19 AR patients were confirmed by a positive skin prick test (SPT) or specific immunoglobulin to Dermatophagoides pteronyssinus and/or Dermatophagoides farinae as well as typical symptoms. The 19 healthy controls had no history of allergic symptoms, and the allergen test was negative. Subjects with usage of systemic corticosteroids in the previous one month, smoking history, immune diseases, pregnancy, or lactation were excluded. Our study was approved by local ethical committee boards, and written informed consent was obtained (Approval number 20190134).

### 2.2. Clinical Severity

A total nasal symptom score (TNSS) was obtained by scoring the symptoms such as nasal obstruction, itchy nose, sneezing, and runny nose from a 0 to 3 scale (0, none; 1, mild; 2, moderate; and 3, severe). The scores were averaged in an 8-week observation period as described in a previous study [17].

### 2.3. Detection of ILC2 from Peripheral Blood Mononuclear Cells by Flow Cytometry

Peripheral blood mononuclear cells (PBMCs) were enriched by centrifugation using Ficoll gradients. Isolated PBMCs (1∗10^6^/mL) were reactivated by phorbol myristate acetate (PMA) (50 ng/mL), ionomycin (500 ng/mL) for 4 hours, and Brefeldin A for 3 hours as described in a previous study [18]. Then, the PBMCs were stained using a lineage-negative cocktail kit (eBioscience, San Diego, CA), CRTH2^+^, and CD127^+^ (BD Bioscience, NJ). Cells were analyzed by a Beckman flow cytometer (Beckman Coulter, CA, USA).

The recombinant IL-25 (10 ng/mL), IL-33 (10 ng/mL), thymic stromal lymphopoietin (TSLP) (10 ng/mL), and IL-2 (50 ng/mL) were added in the medium for ILC2 stimulation.

### 2.4. Isolation of ILC2

The lineage-negative (Lin^−^) cells were enriched from PBMCs by an EasySep Fluorescein Isothiocyanate (FITC) selection kit. Then, the Lin^−^ cells were stained with PE-labeled CRTH2 (BM16, BD Bioscience, NJ) and PE-Cy7-labeled CD127 (HIL-7R-M21, BD Bioscience, NJ) for sorting of ILC2s (Lin^−^CRTH2^+^CD127^+^) by a MoFlo XDP cell sorter (Beckman Coulter, CA, USA). The fluorescence minus one (FMO) (1 antibody against CRTH2 or CD127 was individually omitted) and isotype controls were also prepared. The sorted ILC2s (1.5 × 10^5^ cells/mL) were cultured in RPMI-1640 with 10% FBS and 1% penicillin/streptomycin supplemented with IL-25 (10 ng/mL), IL-33 (10 ng/mL), TSLP (10 ng/mL), and IL-2 (50 ng/mL) for 3 days.

Various concentrations of IL-27 (10-100 ng/mL), anti-IL-27 (100 ng/mL), and IL-10 (100 ng/mL) were provided in some experiments described in a previous study [15]. All stimulators and inhibitors were purchased from R&D Systems. The ILC2 proliferation was measured by using tritiated thymidine incorporation. The expression of gp130 and WSX-1 by ILC2 was detected by staining of the antibody to gp130 and WSX-1 (BD Bioscience, NJ) using flow cytometry.

### 2.5. Quantitative Real-Time Polymerase Chain Reaction (qRT-PCR)

The total RNA from ILC2s was obtained using a TRIzol reagent (Life Technologies, California). The RNA (1 *μ*g) was reversely transcribed using a cDNA kit (QIAGEN). The cDNA was synthesized using an Oligo(dT)_12–18_ Primer and SuperScript III Reverse Transcriptase (Life Technologies). PCR was carried out using an ABI PRISM 7300 Detection System. The expression target genes were calculated using the change-in-cycling-threshold (Ct) method and normalized to the housekeeping gene as described in a previous study [19].

### 2.6. Enzyme-Linked Immunosorbent Assay (ELISA)

The concentrations of cytokines from the supernatant were detected using ELISA kits (R&D Systems, USA) as described by instructions. The detection sensitivity for cytokines was listed as follows: IL-5, 3.9 pg/mL; IL-13, 125 pg/mL; and IL-27, 156 pg/mL.

### 2.7. Animal Model

Six- to eight-week-old BALB/cJ mice (30 mice) were raised in a specific pathogen-free animal facility provided with food and water. On day 0 and day 7, the mice were sensitized with 100 *μ*g of Der p1 and 2 mg of aluminium hydroxide intraperitoneally. Intranasal challenge of Der p1 (100 *μ*g) was done on days 15, 16, and 19. The control mice received intranasal PBS or IL-27 (0.5 *μ*g, R&D Systems) 1 hour before rechallenge with Der p1. Nasal tissue was collected and prepared for further experiments. All animal care and experimental protocols were approved by local ethics committee boards.

### 2.8. ILC2 Detection in Mouse Model

Cells from nasal tissue were stained with Lin^−^ (TCR*β*, TCR*γδ*, CD3*ε*, Fc*ε*R1*α*, Gr-1, CD11b, TER-119, B220, NK1.1, CD5, and CD11c), ST2, CD45, CD127, KLRG1, and Thy-1. The ILC2s were defined as Lin^−^ ST2^+^CD45^+^CD127^+^KLRG1^+^Thy-1^+^cells. The IL-5- and IL-13-positive ILC2 cells were stained using Cytofix (BD Bioscience, NJ) as described by the manufacturer's instructions.

### 2.9. Statistical Analysis

Statistical analysis was performed by SPSS 11.0. The Kruskal-Wallis *H* test or the nonparametric Mann-Whitney *U* test was done. Correlations were analyzed by the Spearman rank correlation analysis. A *P* value of less than 0.05 was considered as statistically significant.

## 3. Results

### 3.1. The IL-27 Serum Levels and Their Correlation with ILC2 and Clinical Severity in AR

**Figure 1:**
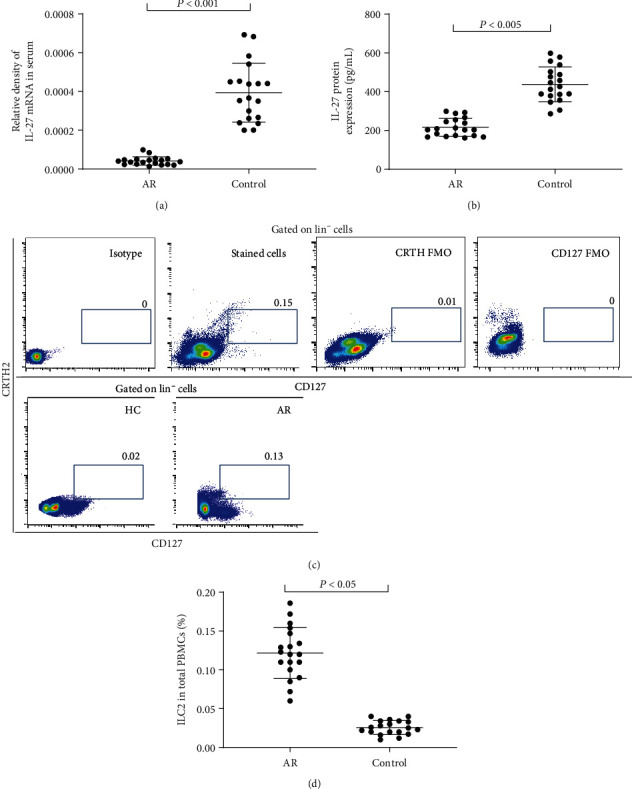
The serum expression of IL-27 and the proportion of ILC2 in PBMCs between AR and controls. (a, b) Expression of serum IL-27 mRNA and protein levels between AR and controls by polymerase chain reaction and enzyme-linked immunosorbent assay, respectively. (c) Frequency of ILC2 in PBMCs between controls and AR as well as isotype and FMO controls by flow cytometry. AR: allergic rhinitis; FMO: fluorescence minus one; HC: healthy control; ILC2: group II innate lymphoid cells; PBMC: peripheral blood mononuclear cells.

**Figure 2:**
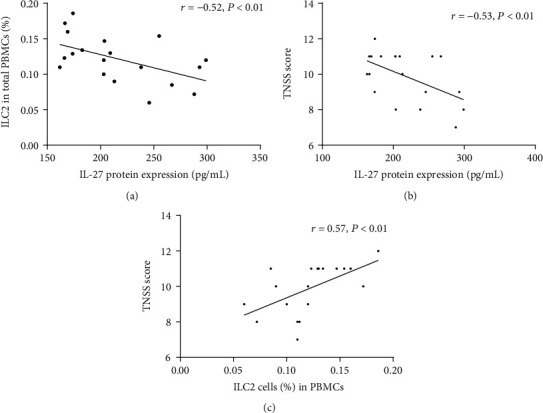
Correlation between of IL-27 protein expression, the proportion of ILC2 in PBMCs, and TNSS score in AR. (a, b) Correlation between IL-27 protein levels and ILC2 frequency and TNSS score. (c) Correlation between ILC2 frequency and TNSS score. AR: allergic rhinitis; ILC2: group II innate lymphoid cells; PBMC: peripheral blood mononuclear cells; TNSS: total nasal symptom score.

The characteristics of participants are summarized in Table 1. To explore the correlation between IL-27 and the proportion of ILC2 as well as clinical severity, we measured the mRNA and protein levels of serum IL-27 and found that they were downregulated in AR patients compared with controls (Figures 1(a) and 1(b)), whereas the proportion of ILC2 in PBMCs was significantly elevated in AR patients compared with controls (Figures 1(c) and 1(d)). The IL-27 protein levels were negatively correlated with the proportion of ILC2 (*r* = −0.52, *P* < 0.01) and TNSS score (*r* = −0.53, *P* < 0.01) (Figures 2(a) and 2(b)), whereas the proportion of ILC2 was positively correlated with TNSS score (*r* = 0.57, *P* < 0.01) (Figure 2(c)).

### 3.2. The ILC2 Cell Regulation by IL-27

**Figure 3:**
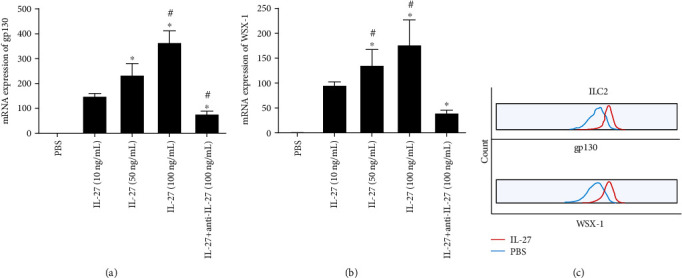
The IL-27 receptor expression by ILC2 regulated by IL-27. (a, b) The mRNA expression of gp130 and WSX-1 by ILC2 after IL-27 stimulation by polymerase chain reaction. (c) The protein expression of gp130 and WSX-1 by ILC2 after IL-27 stimulation by flow cytometry. Three independent tests were performed for every experiment. ^∗^Compared with IL-27 (10 ng/mL), *P* < 0.05. ^#^Compared with IL-27 (50 ng/mL), *P* < 0.05. gp130: glycoprotein 130; ILC2: group II innate lymphoid cells.

To explore the direct interaction between IL-27 and ILC2, we detected the IL-27 receptor by ILC2 after IL-27 stimulation. Our results showed that the mRNA and protein expressions of the IL-27 receptor (gp130 and WSX-1) by ILC2 were significantly upregulated after IL-27 stimulation (Figures 3(a)–3(c)). However, IL-27 receptor expression by healthy control and patient samples under steady state was undetectable.

**Figure 4:**
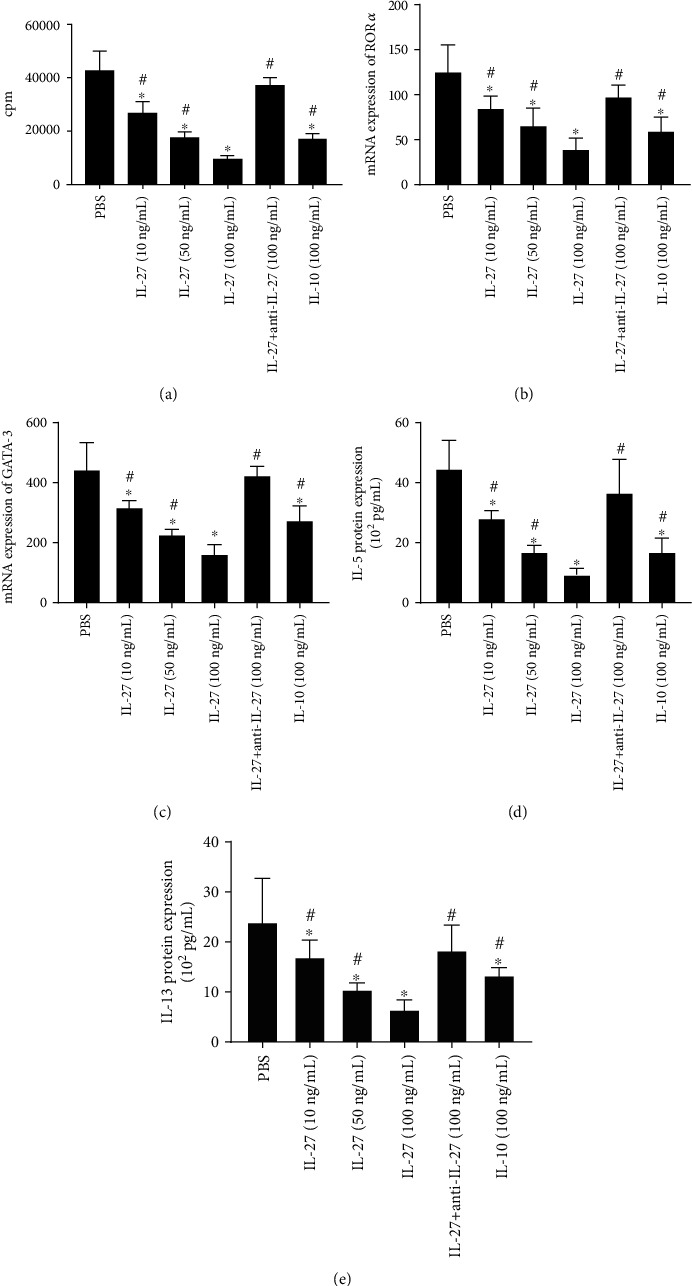
IL-27 inhibited ILC2 cell proliferation and cytokine expression. (a) Proliferation of ILC2 was assessed by tritiated thymidine incorporation under IL-27 stimulation. (b, c) The mRNA expression of GATA-3 and ROR*α* by ILC2 detected by PCR. (d, e) Type II cytokine protein expression by ILCA2 measured by ELISA. Three independent tests were performed for every experiment. ^∗^Compared with PBS, *P* < 0.05. ^#^Compared with IL-27 (100 ng/mL), *P* < 0.05. cpm: counts per minute; ROR*α*: Retinoic Acid-Related Orphan Receptors *α*; GATA-3: GATA binding protein-3; ILC2: group II innate lymphoid cells.

Next, we investigated the proliferation and function of ILC2 regulated by IL-27. We found that IL-27 inhibited the proliferation of ILC2 as well as GATA binding protein-3 (GATA-3) and Retinoic Acid-Related Orphan Receptor *α* (ROR*α*) production by ILC2 directly in a dose-dependent manner (Figures 4(a)–4(c)). Moreover, the production of IL-5 and IL-13 by ILC2 were also inhibited after IL-27 stimulation in a dose-dependent manner (Figures 4(d) and 4(e)).

### 3.3. IL-27 Inhibits Allergic Responses in Mouse Model

**Figure 5:**
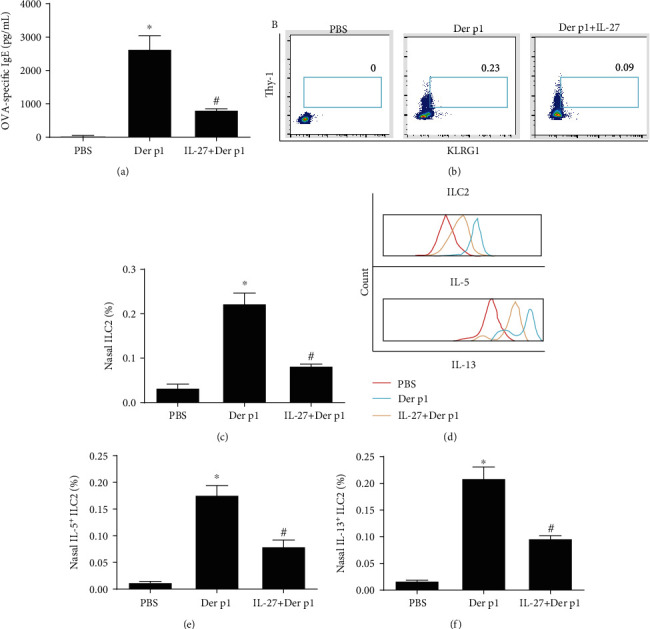
IL-27 inhibits allergic responses in mouse model. (a) Levels of Der p1-specific IgE by enzyme-linked immunosorbent assay after IL-27 stimulation. (b, c) The proportions of nasal ILC2 in total cells of inferior turbinate tissue after IL-27 treatment by flow cytometry. (d–f) The proportions of nasal IL-5+ILC2 and IL-13+ILC2 in total cells of inferior turbinate tissue. After IL-27 treatment by flow cytometry. Ten mice were allocated into every group. Three independent tests were performed for every experiment. ^∗^Compared with PBS, *P* < 0.05. ^#^Compared with Der p1 (100 ng/mL), *P* < 0.05. OVA: ovalbumin; Der p1: dermatophagoides p1 protein; ILC2: group II innate lymphoid cells.

To prove the *in vivo* effect of IL-27, we treated allergic mice with IL-27. The IL-27 reduced the Der p1-specific IgE level (Figure 5(a)) and decreased the proportion of nasal ILC2 in total cells of inferior turbinate tissue (Figures 5(b) and 5(c)) and type II cytokine production (Figures 5(d)–5(f)) by nasal ILC2s compared with control mice.

## 4. Discussion

In this study, our data demonstrated that IL-27 inhibited the proliferation and function of ILC2s, providing a new mechanism for the pathogenesis of AR.

Recent studies suggest that IL-27 has broad effects on Th1, Th2, and Th17 subsets of T cells and regulatory T cells [20, 21]. For example, IL-27 can inhibit Th2 responses in AR patients. In animal models, intranasal administration of IL-27 decreased nasal allergic responses and symptoms even after the establishment of allergic rhinitis [22, 23]. These results suggested a natural protective role of IL-27, of which signaling deficiency induces a Th17-type hyperresponse that further aggravates the Th2-dominant allergic response [24].

For ILC2s, IL-27 has been reported to suppress ILC2 cells in a manner dependent on the transcription factor signal transducer and activator of transcription 1 (STAT1) during lung inflammation induced by Alternaria alternata [15]. Moreover, IL-27 inhibited ILC2 proliferation and cytokine production and significantly inhibited their accumulation in papain-induced airway inflammation [16]. However, no study explored the effect and mechanism of IL-27 on ILC2 in AR. Our in vitro results suggested that IL-27 can inhibit the proliferation and function of ILC2 directly by regulation of IL-27R on ILC2. We first reported a dose-dependent manner of IL-27R expression regulated by IL-27, proving the direct effect of IL-27 on ILC2. Consistently, Mchedlidze et al.'s study also suggested that IL-27 receptor signaling directly regulates ILC2 responses. Our study provided a novel mechanism for the negative regulation of ILC2 immune responses in AR [16].

GATA-3 is needed for the maintenance of mature ILC2 cells as well as their precursors in bone marrow. Moreover, GATA-3 has been shown to be necessary for the production of IL-5 and IL-13 by human ILC2 cells. ROR*α* is also essential for ILC2 development, as ROR*α*-deficient mice lack ILC2 cells [25]. Our data also confirmed that IL-27 inhibited both expressions of GATA-3 and ROR*α*, which contributed to the decreased proliferation and cytokine production by ILC2.

Similarly, the allergic responses in the mouse model were significantly abrogated when IL-27 were added and presented with reduced Der p1-specific IgE level and decreased proportion of nasal ILC2 and type II cytokines. Consistently, Mchedlidze et al.'s study demonstrated that IL-27 deficiency is correlated with increased mucosal infiltration of ILC2 in an inflammatory lung disease model. Moreover, IL-27 treatment inhibited ILC2 proliferation and cytokine production and significantly inhibited their accumulation in vivo [16].

In this study, we used human ILC2 to investigate the effect of IL-27 on ILC2 in vitro. However, it needed further studies to prove if IL-27 suppresses AR in vivo in humans. In summary, our study proved the effect of IL-27 in the regulation of ILC2s through in vitro and in vivo studies. Our results implied that IL-27 may be used as a promising treatment target in AR.

## Figures and Tables

**Table 1:** Demographic characteristic of AR patients and normal controls.

Groups	AR group	Control
Number	19	19
Sex (male : female)	10 : 9	9 : 10
Age (years)	23.6 (18-42)	22.1 (18-46)
TIgE (kU/L)	357.2 (68.4-718.9)^∗^	34.2 (6.8-71.8)
TNSS score	8.1 (6-12)	—

^∗^Compared with control group, *P* < 0.05. AR: allergic rhinitis; TNSS: total nasal symptom score.

## Data Availability

The datasets used and/or analyzed during the current study are available from the corresponding author on reasonable request.
